# Mainstreaming biodiversity and wildlife management into climate change policy frameworks in selected east and southern African countries

**DOI:** 10.4102/jamba.v8i3.254

**Published:** 2016-04-08

**Authors:** Olga L. Kupika, Godwell Nhamo

**Affiliations:** 1 Institute of Corporate Citizenship, Exxaro Chair in Business & Climate Change, University of South Africa, South Africa; 2 School of Wildlife, Ecology and Conservation, Chinhoyi University of Technology, Zimbabwe

## Abstract

The Rio+20 outcomes document, the Future We Want, enshrines green economy as one of the platforms to attain sustainable development and calls for measures that seek to address climate change and biodiversity management. This paper audits climate change policies from selected east and southern African countries to determine the extent to which climate change legislation mainstreams biodiversity and wildlife management. A scan of international, continental, regional and national climate change policies was conducted to assess whether they include biodiversity and/or wildlife management issues. The key finding is that many climate change policy–related documents, particularly the National Adaptation Programme of Actions (NAPAs), address threats to biodiversity and wildlife resources. However, international policies like the United Nations Framework Convention on Climate Change and Kyoto Protocol do not address the matter under deliberation. Regional climate change policies such as the East African Community, Common Market for Eastern and Southern Africa and African Union address biodiversity and/or wildlife issues whilst the Southern African Development Community region does not have a stand-alone policy for climate change. Progressive countries like Rwanda, Uganda, Tanzania and Zambia have recently put in place detailed NAPAs which are mainstream responsive strategies intended to address climate change adaptation in the wildlife sector.

## Introduction

Climate change poses challenges for both natural and human ecosystems. This state of affairs calls for sectoral interventions at international, continental, regional and national scale in order to protect and preserve biodiversity, particularly wildlife (IPCC 2001). Chuku (2010) claims that climate change concerns do not feature prominently in the implementation of national and regional development programmes, although this has been part of the global agenda for sustainable development since 1992 when the Rio Conference was held in Brazil. Countries around the globe made a commitment to tackle this global challenge through signing and ratification of the 1992 United Nations Framework Convention on Climate Change (UNFCCC) (Sokona & Denton 2001). Consequently, the Kyoto Protocol was adopted in 1997 at the 3rd Conference of Parties (COP) (UNFCCC 1997). Thus, global climate change policy through UNFCC and its Kyoto Protocol is aimed at linking the issues of greenhouse gas emissions (GHG) and development (Stringer *et al*. 2009). The UNFCC works in conjunction with a number of other agencies and partner organisations related to different sectors of the environment.

In pursuit of the then Millennium Development Goals (MDGs) to achieve sustainable development, individual countries have made an effort to make the climate change issue a vital component of long-term policy and planning (Sokona & Denton 2001). In 2012, the Rio+20 agenda, in pursuit of sustainable development goals, made calls for measures that seek to address climate change and biodiversity management issues within the green economy transition as one of the enabling platforms (Nhamo 2014a). However, since the inception of the UNFCCC in 1992 and consequently the Kyoto Protocol in 1997, global discussions on climate change have not seriously considered biodiversity, particularly wildlife (Van Dyke 2008) as it pertains to the impact climate disaster risk has on the sector.

Following the Rio+ 20 Summit held in Brazil in June 2012, global leaders recognise the need to support mainstreaming the consideration of the socio-economic impacts and benefits of the conservation and sustainable use of biodiversity and its programmes and policies at all levels, in accordance with national legislation, circumstances and priorities (United Nations Environment Programme [UNEP] 2012a:36). The United Nations is formally committed to biodiversity mainstreaming in all environment-related policies and programmes, including policy-making processes relating to climate change (Heller & Zavaleta 2009). Nevertheless, biodiversity-related aspects are rarely addressed in climate change policies either at the national or at the international levels. Attempts to link climate change and biodiversity have been discussed in the Intergovernmental Panel for Climate Change Technical Paper V on climate change and biodiversity (Suárez, Watson & Dokken 2002). The paper highlights the need to identify biodiversity conservation and sustainable use activities and policies that would beneficially affect climate change adaptation and mitigation options (Van Dyke 2008).

**FIGURE 1:**
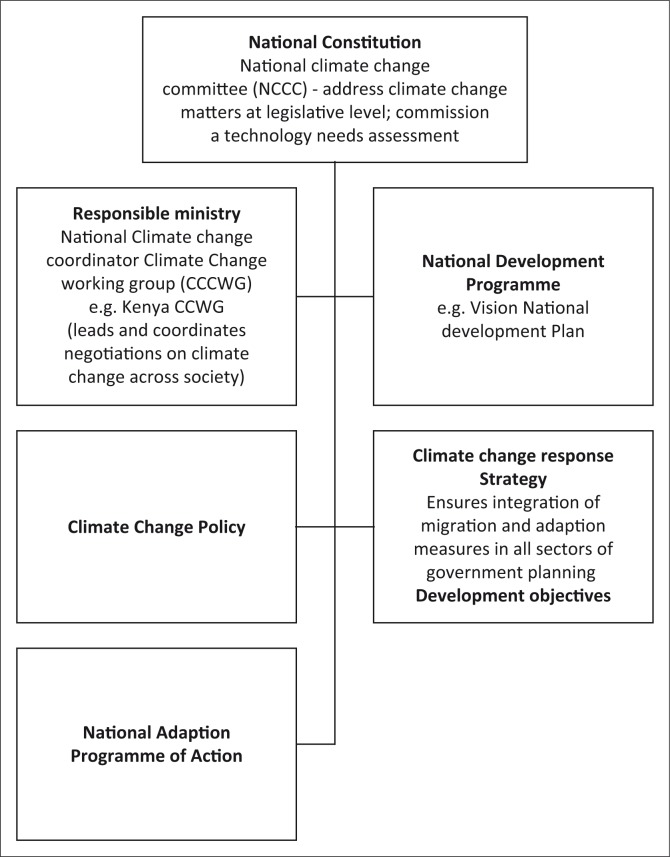
Representation of national climate change policy frameworks.

The UNFCCC requires that each Least Developed Country (LDC) should develop and implement a National Program of Action (NAPA) as part of climate change policy framework, which aims at addressing the climate change agenda (AWEPA 2012). From the Institute of Development Studies (IDS) (2000), mainstreaming implies that awareness of biodiversity and/or wildlife management is integrated into the existing and future climate change policies and plans at the international, regional and national levels. Generally, countries in east and southern Africa have adopted the following framework for developing climate change policies (Figure 1).

This paper investigates how climate change policy frameworks (inclusive of laws, regulations, whitepapers, plans, strategies, directives, etc.) mainstream biodiversity and/or wildlife management issues in selected east and southern African countries. In this paper, we assume that the strategies for monitoring the impacts of climate change in African protected areas are based on an enabling climate change policy framework. To date, most studies have analysed the implications of policy and legal mechanisms on climate change in key sectors such as agriculture, water, energy and tourism and the environment in general. However, few studies have focused on how international, regional and national climate change policy mainstream biodiversity and/or wildlife management. There is therefore need to review the implication of climate change policy intervention towards biodiversity management in the face of climate change.

This review comes in three sections. The first section deals with the methodology followed by literature review on climate change and wildlife. The second section examines how international, regional and selected national climate change policies mainstream biodiversity and/or wildlife management. The last section highlights conclusions and recommendations on how countries in east and southern Africa are addressing critical and increasing climate threats in the wildlife sector.

## Climate change and wildlife management in Africa

This section sets the scene by defining concepts and issues related to climate change and wildlife management in Africa. Wildlife management refers to the process by which wildlife is kept within desirable levels through the use of relevant scientific, technical and traditional methods (Morgera & Wingard 2009). Taking this further, wildlife management is therefore concerned with activities such as species monitoring and surveys, problem animal control, cropping, culling, capture and introductions, wildlife diseases and pests (GNP 2010). The key components in wildlife management include international and regional treaties and conventions, national parks, national legislations and policies, nongovernmental organisations (NGOs), private operators and local communities living adjacent to protected areas. In this review, the term ‘biodiversity’ will be used interchangeably with wildlife depending on the context of the policy document. A preliminary scan of international policy documents revealed that the term ‘biodiversity’ is mostly used in international and regional conservation policy. However, at the national and local scale, particularly in most African countries, for management purposes, the term ‘wildlife’ is adopted as a key component of biodiversity.

The IPCC (2007) defines climate change as:

any change in climate in the state of the climate that can be identified by changes in the mean and/or the variability of its properties and that persists for an extended period typically over decades or longer. (p. 30)

Climate change may be caused by natural changes or persistent anthropogenic changes in the composition of the atmosphere or land use (USAID 2007). The UNFCCC defines climate change as a change of climate which is attributed directly or indirectly to human activity that alters the composition of the global atmosphere and which is in addition to natural climate variability observed over comparable time periods (African Ministerial Conference on Environment [AMCEN] 2011). Climate variability is defined as the variations in the mean state and other climate statistics on temporal scales beyond those of individual weather events. It may result from natural internal processes within the climate system or from variations in natural or anthropogenic external variability (AMCEN 2011; IPCC 2007).

## Methodology

This study aimed to answer the following question: to what extent do east and southern Africa climate change policies mainstream matters relating to sustainable biodiversity conservation and wildlife management? This review focused on selected countries from east and southern Africa. The countries purposefully sampled include Botswana, Kenya, Malawi, Namibia, Rwanda, South Africa, Tanzania, Uganda, Zambia and Zimbabwe. The countries were sampled based on a number of factors, amongst them, the viability of the wildlife sector, those that are English speaking and the availability of the necessary documents online.

The study is mainly literature based, focusing on a number of climate change policy documents from a range of international, continental, regional policy frameworks, protocols and declarations as well as national policies that have been developed in the sample countries. Most policies were developed after the 1992 Rio Declaration when the climate change agenda became an international concern. The retrieved and examined legislation is presented in Table 1. The authors examined climate change–related policy documents to determine the extent to which they incorporate wildlife issues. It emerged in the preliminary analysis that generally many global treaties and conventions such as the Convention on Biological Diversity (CBD) do not specifically mention the term ‘wildlife’. To this end, we assumed that reference to biodiversity includes wildlife. Climate change–related legislation was therefore examined following key phrases or words related to wildlife management: ‘wildlife’, ‘wild species’, ‘biodiversity’, ‘biological diversity’, ‘plants and/or animals’, ‘flora and/or fauna’. In this review, we did not carry out a detailed analysis or comparison of policies.

**TABLE 1:** Sample regions and countries’ policy documents related to climate change.

Country or region	Policy	Year
Global Area	UNFCCC	1992
	Kyoto Protocol	1997
Continental (Africa)	Draft African Climate Change Strategy	2014
Common Market for Eastern and Southern Africa (COMESA)	COMESA Climate Initiative	2007
East African Community (EAC)	EAC Climate Change Policy (2011)	2011
	EAC Climate Change Master Plan (2011–2031)	2011
	EAC Climate Change Strategy	2011
Southern Africa Development Corporation (SADC)	Draft SADC Climate Change Programme	2010
Botswana	Constitution of Botswana	1994
	National Development Plan 10 (2009–2016)	2009
	Vision 2016	2009
	Botswana National Action Plan (2011–2016)	2011
Kenya	The Constitution of Kenya	2010
	National Climate Change Response Strategy (NCCRS)	2012
	Draft Climate Change Framework Policy	2014
Malawi	The Constitution of Malawi	1994
	Malawi’s National Adaptation Programmes of Action (NAPAs)	2006
Namibia	The Constitution of the Republic of Namibia	1998
	National Policy on Climate Change for Namibia	2010
	Proposed Climate Change Strategy & Action Plan	2009
Rwanda	Rwanda’s Constitution of 2003 with Amendments through 2010	2010
	National Strategy for Climate Change and Low Carbon Development	2011
South Africa	The Constitution of the Republic of South Africa	1996
	National Climate Change Response Strategy White Paper	2012
	National Development Plan: Vision 2030	2011
Tanzania	The Constitution of the United Republic of Tanzania	1997
	Tanzania National Climate Change Strategy	2013
	National Adaptation Programme of Action	2007
Uganda	Constitution of the Republic of Uganda	2006
	Draft Uganda National Climate Change Policy 2012	2012
	Uganda Vision 2040	2000
	National Development Plan	2013
	Ugandan National Programme of Action	2010
Zambia	The Constitution of Zambia (amendment) Bill	2015
	National Climate Change Response Strategy (NCCRS)	2010
	Report on formulation of the National Adaptation Programme of Action on Climate Change	2002
Zimbabwe	Constitution of Zimbabwe	2013
	Zimbabwe’s National Climate Change Response Strategy	2015
	*Environmental Management Act* 13 of 2002, chapter 20:27	2002

**FIGURE 2:**
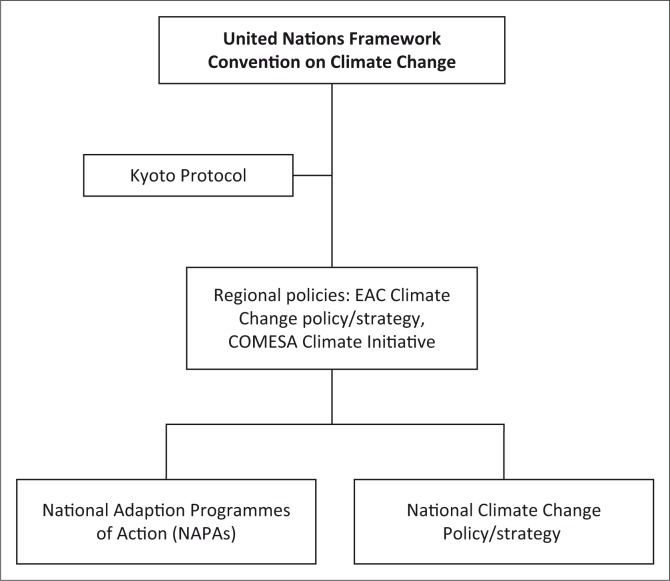
Framework for incorporation of wildlife management issues in climate change policy.

For each policy document, instances where wildlife management issues are mentioned were briefly highlighted and discussed. Data were then examined and interpreted to deduce meaning and draw conclusions leading to the development of empirical knowledge (Nhamo 2014a). The examination process followed the general international, regional and national legal and institutional policy framework for climate change policy (Figure 2).

## Results and discussion

This section highlights the key findings from the study. We present findings on how international, continental and regional as well as national climate change policies mainstream biodiversity and wildlife management issues.

### International and regional climate policies and biodiversity and wildlife management

The UNFCCC and the Kyoto Protocol are the key international instruments which address climate change (Mburia 2015; UNFCCC Secretariat 2008). Provisions for inclusion of biodiversity and wildlife–related aspects are highlighted in Table 2.

**TABLE 2:** Biodiversity and wildlife management–related provisions in international and regional climate change policies.

Treaty or convention	Year	Comment
UNFCCC	1992	No direct mention of biodiversity and wildlife-related terms
Kyoto Protocol	1997	No direct mention of biodiversity and wildlife-related terms
Draft African Climate Change Strategy	2014	Mentions biodiversity (see detailed table)
EAC Climate Change Master Plan (2011–2031)	2011	Conservation and sustainable use of wildlife
EAC Climate Change Policy (EACCCP)	2012	Sectoral conservation and sustainable use of wildlife
EAC Climate Change Strategy	2013	Protection of wildlife and key vulnerable ecosystems
The 4th EAC Development Strategy (2015/2016)	2012	No direct mention of wildlife
Draft SADC Climate Change Programme	2010	No direct mention of wildlife
COMESA Climate Initiative		No direct mention of wildlife issues
Programme on Climate Change Adaptation and Mitigation in the Eastern and Southern Africa (COMESA-EAC-SADC) region	2011	Mentions biodiversity

Although the UNFCCC and the Kyoto Protocol do not directly mention biodiversity and wildlife–related terms, the United Nations under its subsidiary organisations, that is UNEP and the United Nations Development Programme (UNDP), has been committed to addressing environmental challenges across the globe. The UNEP conducts complementary programmes and initiatives to encourage the development and implementation of environmental law by states. UNEP’s work on climate change is shaped by the negotiations process of the UNFCCC (UNEP 2011). However, UNEP is also scaling up its role and response to climate change under three priority areas that match calls for international guidance, the urgent need for action at a national level on climate change and the organisation’s skill set, experience and mandate, that is Ecosystem-Based Adaptation (Eba), Reducing Emissions from Deforestation and Forest Degradation plus (REDD+) and Clean Technology (UNEP 2011).

The UNDP emphasizes the need for Eba to biodiversity conservation (UNEP 2012b). A technical paper on interlinkages between climate change and biodiversity, released in 2002, was prepared at the request of the CBD (IPCC 2001). The CBD has been actively involved in the UNFCCC process, exchanging information and expertise on activities and outcomes from their processes (UNEP 2012b). Despite the existence of such institutions and policies, the Rio conventions, particularly the UNFCCC, are yet to enhance synergies with biodiversity constituencies and countries are yet to come up with a binding agreement at 3rd Conference of Parties 21 in Paris, which will also take into consideration the role of healthy, resilient ecosystems in addressing climate change mitigation agenda (UNEP 2012b).

### African union climate change policy and biodiversity

The African Union (AU) under the AMCEN has the mandate to develop climate change adaptation and mitigation policy as well as frameworks for low carbon development technology (Mburia 2015). The AMCEN provides political guidance and monitoring to the development of Africa’s position towards climate change adaptation policy formulation into specific national states. However, the AU does not have a stand-alone climate change policy (Mburia 2015). The region has several strategic climate change working papers under the 2003 AU-NEPAD Environment Action Plan (EAP) (Mburia 2015). Although the document includes eight sectoral priority areas including the environment and programmes related to the environment in general, part of the agenda mentions biodiversity. For instance, the initiative notes that ‘climate change is the latest threat to biodiversity in Africa’ (AMCEN 2014:74). In 2014, the region developed the draft African Climate Change Strategy (AMCEN 2014). Section XI.10 of this document focuses on biodiversity and ecosystems. The strategy stipulates that ‘biodiversity degradation largely occurs outside protected areas and is under threat from habitat loss and over-exploitation of natural resources, amongst others, whose effects are exacerbated by climate change’ (AMCEN 2014:41–42). A summary reflecting how biodiversity mainstreaming in the draft African Climate Change Strategy will take place is shown in Table 3.

**TABLE 3:** Summary of biodiversity mainstreaming in African climate change strategy.

Section	Biodiversity mainstreaming comments or provisions
XI.10 Biodiversity and Ecosystems	Biodiversity degradation largely occurs outside protected areas and is under threat from habitat loss and over-exploitation of natural resources, among others, whose effects are exacerbated by climate change (p. 74).
Goal 9 AU to provide leadership through its specialized technical committees (STCs) in the negotiation and implementation of CBD, United Nations Convention to Combat Desertification and other biodiversity-related conventions and agreements	Action 1: Provide support for the implementation and tracking of the various decisions by AU, AMCEN biodiversity (CBD), and land degradation (United Nations Convention to Combat Desertification) among others. Action 2: Provide required support for developing or improving frameworks for implementation of climate resilient biodiversity management within AU Member States, RECs and other regional relevant bodies. Action 3: Provide leadership in the development of a framework for capacity building, training, research and communication on climate resilient biodiversity preservation and conservation. Action 4: Support development, improvement and harmonisation of common policies, laws and strategies relating to biodiversity, land desertification etc. including trans-boundary issues for the conservation and sustainable utilization of biodiversity in and outside protected areas (p. 42).
Goal 35: Enhance the integration of climate change resilience in the development plans and programmes of Small Island Developing States (SIDS)	Action 5: Address island biodiversity in a manner that responds to the unique characteristics of SIDS (p. 56)
IX.5. Adaptation, mitigation, and the green economy	Green economy system allows for the normal functioning of the life support functions, including the source and waste functions, in a way that reinforces the ecological integrity of biological processes, and, in the main, ensures the continued existence of viable natural assets for the overall improvement of welfare of biodiversity in all its forms. (p. 25)

*Source:* Adapted from AMCEN, 2014, *African strategy on climate change*, AMCEN Secretariat, Addis Ababa, Ethiopia

### East African Community climate change policy and biodiversity

The East African Community (EAC) Climate Change Policy is complementary to various international conventions and protocols related to climate change and biodiversity management such as the UNFCCC and Kyoto Protocol. The policy is further linked to other multilateral environmental agreements such as the United Nations Convention to Combat Desertification (UNCCD), CBD, Convention on International Trade in Endangered Species (CITES) and the Ramsar Convention on Wetlands. Chapter 2 of the EAC Climate Change Policy contains policy statements and actions under sectoral planning and implementation of climate change adaptation measures in key sectors such as wildlife and tourism (EAC Secretariat 2010). A summary of the provisions is highlighted in Box 1.

**BOX 1:** Summary of sectoral provision for wildlife in East African Community Climate Change Policy.

**Wildlife** The great reservoir of East Africa’s wildlife and biological diversity is increasingly under threat as a result of ecosystem fragmentation, over utilization of resources and conflicts between wildlife and other human activities such as agriculture and human settlement. Persistent drought due to increase in temperature and unreliable rainfall pattern in the region is expected to affect the lifestyles of most of the migratory wild species, in particular the wildebeest and some bird species. The wildlife forms an important source of food and income for some local communities in the region. Change in ecological systems will lead to disappearance of some wild animal species.
**Sectoral challenges** The Sectoral challenges facing wildlife in light of climate change include: ecosystems change (in terms of biodiversity and climatic conditions) leading to ecological range shifts of specific species destruction of wildlife habitats due to increased natural bush fires decreasing carrying capacity of Protected Areas (PAs) and rangelands due to increasing extreme weather conditions, leading to reduced regeneration of pastures and water resources for the wild animals.
**Sector specific objective** To develop, harmonize and adopt common policies, laws and strategies for the conservation and sustainable utilization of wildlife resources in and outside protected areas in the region.
**Sectoral Policy statements** East African Community Partner States shall: livelihood for local communities in order to reduce their dependency on wildlife promote measures that preserve the ecosystem integrity of critical wildlife habitats and endangered species establish, promote, and/or protect wildlife migration corridors.
**Tourism sector objective and policy statements** To ensure resilience of tourism infrastructure through factoring Climate Change Climate Change into their planning, as well as enhancing climate proofing of wildlife habitats to minimize environmental migrations of endangered species. Develop all weather infrastructure to support tourism in the region while ensuring minimal damage to wildlife habitats. Develop park management practices which will enable wildlife to adapt to the changing climate.

*Source:* EAC Secretariat, 2010, *EAC Climate Change Policy (EACCCP)*, p. 10, viewed 09 April 2015, from http://www.eac.int/environment

Under cross-cutting actions, the EAC Climate Change Policy specifies the need for research and development which ‘promote periodic climate change related research and exchange of information in conservation and sustainable use of wildlife’, (EAC Secretariat 2010:24). The policy also highlights the need for information management and sharing through the development of ‘a database and information sharing system for purposes of monitoring of wildlife resources in the region’, (EAC Secretariat 2010:24). A Strengths Weaknesses Opportunities and Threats analysis of the ecosystems and biodiversity section in the EAC Climate Change Master Plan reveals that one of the strengths is that the region has ‘good policies and institutions for wildlife management’, (EAC 2011a:96). In response to the sectoral challenges highlighted in the EAC Climate Change Strategy, the EAC Climate Change Master Plan (EAC 2011a) includes key strategic interventions for the wildlife sector as indicated in Table 4.

**TABLE 4:** East African Community climate change master plan strategic interventions for the wildlife sector.

Strategic objective	Strategic interventions
1.3 To develop, harmonise and adopt common policies, laws and strategies for the conservation and sustainable utilization of wildlife resources in and outside protected areas in the region as part of ecosystem-based adaptation.	Promote measures that preserve the ecosystem integrity of critical wildlife habitats and endangered species; Establishment of more ‘protected’ areas; collection and conservation of genetic resources of neglected indigenous species. *Protection and management of rare plant and animal species, particularly those of important value, e.g. medicinal; Research and active management of enclosed/protected/marginal areas specifically in relation to determining the carrying capacity and consequently the provision of water pans and other infrastructure to support wildlife in those areas. *Breeding new plant species that are more tolerant to changed climatic conditions. Protecting and enhancing migration corridors and habitat connectivity (e.g. through avoiding habitat fragmentation especially with regard to privately owned land) to allow species to migrate as the climate changes. Facilitate the diversification of livelihood for local communities in order to reduce their dependency on wildlife. *Protection of sensitive ecosystems through measures such as community-driven ecosystem management particularly as a way of addressing the drivers of over-exploitation and degradation of key ecosystems. Facilitate the development of rapid response teams in order to respond to imminent and current destructive activities to limit damage to ecosystems. Promoting fire suppression practices in the event of increased fire risk because of temperature increases. Controlling insect and disease outbreaks. Facilitate the rehabilitation and restoration of degraded habitats/ecosystems.

*Source:* EAC, 2011b, *East African community climate change strategy*, EAC, Arusha, p. 160

**FIGURE 3:**
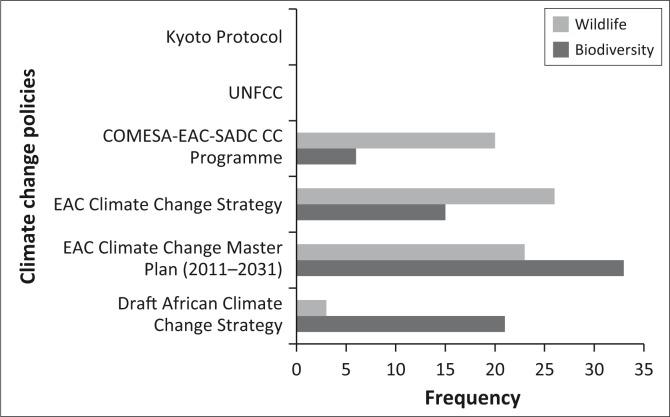
Frequency counts for ‘wildlife’ and ‘biodiversity’ in international and regional climate change policies.

This review shows that the continental and EAC, Common Market for Eastern and Southern Africa-COMESA-EAC-Southern African Development Community (SADC) regional policies for climate change address biodiversity/wildlife management issues. The EAC had detailed provisions on how biodiversity issues should be mainstreamed in the implementation of climate change policies. However, the SADC region does not have stand-alone policies which mainstream climate biodiversity/wildlife issues. Figure 3 shows the frequency counts of the terms ‘biodiversity’ and ‘wildlife’ in international, continental and regional climate change policies.

The AU has made progress in addressing biodiversity issues in the draft African Strategy on Climate Change. The EAC Climate Change Master Plan has the highest frequency of the term ‘biodiversity’ (33), whilst the EAC Climate Change Strategy has the highest count for ‘wildlife’ (26). The COMESA-EAC-SADC climate change policies have low counts of the term ‘biodiversity’ and ‘wildlife’, whilst key international climate change policies, that is the UNFCCC and Kyoto Protocol, do not even mention the terms.

### National climate change policies and wildlife management

**FIGURE 4:**
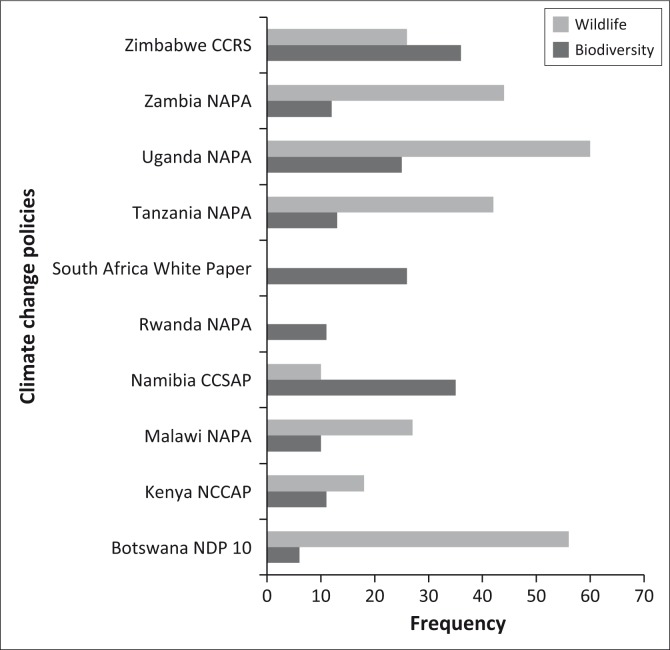
Frequency counts for ‘wildlife’ and ‘biodiversity’ in national climate change policies.

East and southern African countries have developed national climate policies and NAPAs in line with the recommendations from the UNFCCC. To establish whether national climate change policies mentioned ‘biodiversity’ and ‘wildlife’, a scan was completed of selected policy documents. An analysis was then done using advanced search for Microsoft Word 2007 to get simple frequency counts using the terms ‘biodiversity’ and ‘wildlife’ (Figure 4). Generally, there is a higher incidence of citations related to ‘wildlife’ than to ‘biodiversity’ within the selected national climate change policies. For instance, all the selected countries, save for Namibia, South Africa and Zimbabwe, have higher counts of the term ‘wildlife’ as compared to biodiversity (Figure 4). The Ugandan National Programme of Action (2010) has the highest frequency for ‘wildlife’ followed by the Botswana National Development Plan 10 (2009–2016) (Government of Botswana 2009), whereas the South African National Climate Change Response White Paper (DEA 2011) and Rwanda National Adaptation Programmes to climate change (Ministry of Lands, Environment, Forestry, Water and Mines 2006) do not even mention wildlife. On the other hand, Zimbabwe National Climate Change Response Strategy (NCCRS) (Government of Zimbabwe 2014) and Namibia Climate Strategy and Action Plan (CCSAP) (Mfune *et al*. 2009) have almost equal counts for the term ‘biodiversity’, that is 36 and 35, respectively, whilst Botswana National Development Plan 10 (2009–2016) (Government of Botswana 2009) has the lowest count of 6. Overall, all the selected policies mention the term ‘biodiversity’.

Climate policies are coordinated by designated units within respective ministries. For example, in Zimbabwe, the National Steering Committee on Climate Change was established in 1996 to assist the government in designing of climate change policies and coordination of specific climate change projects (Chagutah 2010). The country also signed and ratified the UNFCCC in 1992 and the Kyoto Protocol in 2009 (Brown *et al*. 2012). According to Stringer *et al*. (2009), the UNFCCC requires least developed countries to undertake NAPAs as policy frameworks dedicated to the identification and prioritisation of critically important adaptation activities. However, Zimbabwe has not provided such policies. Instead, the country has provided national communications to the UNFCCC with regards to detailing activities that have been undertaken to implement climate change activities. The country submitted its first national communication on impacts and adaptation in the agriculture, forestry water resources and human and health sectors in 1998 (Lotz-Sisitka & Urquhart 2014). In 2009, Zimbabwe began to prepare and submitted its second national communication which included vulnerability and adaptation on ecosystems, human settlements, public health water resources and wildlife (Brown *et al*. 2012). In 2013, Zimbabwe launched the draft NCCRS (Lotz-sisitka & Urquhart 2014), which provides a framework for a comprehensive and strategic approach on aspects of adaptation, mitigation, technology, financing, public education and awareness. The strategy further includes a National Action Plan for Adaptation and Mitigation. The strategy mentions biodiversity and wildlife in several sections as the sector underpinning the tourism industry.

For completeness, a detailed analysis of climate change policies for Uganda and Botswana was done. The selection is based on the fact that these countries had the highest frequency counts of the term ‘wildlife’.

Climate change issues in Uganda are coordinated by the Climate Change Unit, which is hosted by the Ministry of Water and Environment. The Unit was established alongside with the Climate Change Policy Committee (CCPC). The CCPC, which comprises government ministries and departments, provides policy guidelines to the unit. The Uganda National Climate Change Policy (Ministry of Water and Environment 2013) makes explicit reference to the need to mainstream climate change adaptation into the wildlife and tourism sectors. The Policy presents and objects ‘To ensure the conservation of wildlife resources and plan for improved resilience of tourism resources and infrastructure to climate change’ (Ministry of Water and Environment 2013:vii). A summary of the policy response in relation to the wildlife and tourism sector is presented in Table 5.

**TABLE 5:** Uganda National Climate Change Policy and wildlife management.

Sectoral context and challenges	Policy response	Specific strategies
Climate change – especially droughts, unreliable rainfall patterns and increasing temperatures – will affect the habitats of animal and bird species	To ensure the conservation of wildlife resources and plan for improved resilience of tourism resources and infrastructure to climate change	Develop a national wildlife adaptation strategy that includes well-assessed climate change adaptation strategies
Changes in ecosystems will lead to the disappearance of some wild animal species	-	Promote measures that preserve the integrity of ecosystems that provide critical wildlife habitats and host endangered species
Increasing temperatures are also affecting some attractions, such as the ice caps of the Rwenzori Mountains, which are in danger of disappearing	-	Develop park management practices that will enable wildlife to adapt to the changing climate
-	-	Develop weather-resilient infrastructure to support tourism in the region, whilst ensuring minimal damage to wildlife habitats

*Source:* Adapted from the Ministry of Water and Environment, 2013, *Uganda national climate change policy*, Government Printers, Kampala, p. 22

The National Development Plan 10 (NDP) and Vision 2016 are the central policy documents that contain the country’s responses to the challenges posed by climate change (Nachmany *et al*. 2015). The NDPs are grand scheme policy frameworks, which define the country’s medium-term development goals and strategies. The current NDP 10 (2009–2016) plan stipulates that climate change–relevant initiatives and programmes need to be mainstreamed in development processes and in all sectors of the environment such as forestry, energy and including forestry and Community-Based Natural Resource Management Programme (Nachmany *et al*. 2015).

Botswana is currently developing a National Climate Change Policy and Strategy and Action Plan(NCCPSAP). The policy will be implemented through the Ministry of Environment, Wildlife and Tourism in co-operation with the United Nations Development Programme. Amongst other objectives, the NCCPSAP aims to develop and implement appropriate adaptation strategies and actions that will lower the vulnerability of Botswana and various sectors of the economy to the impacts of climate change.

Findings from the other climate change documents (not included in the graphical analysis) revealed that the South African standing legislation for climate change, that is the National Climate Change Response White Paper (DEA 2011), as well as Rwanda Vision 2020 do not even mention the term ‘wildlife’. However, the Zambian NCCRS (Government of the Republic of Zambia 2010) has the highest frequency count of the term ‘wildlife’ (46), followed by the Malawi’s National Adaptation Programmes of Action (Ministry of Mines, Natural Resources and Environment 2006) with 27 counts and then the Zimbabwe NCCRS (Government of Zimbabwe 2014) with 26 counts.

Generally, National Adaptation Programmes of Action and climate change strategies from both the EAC and SADC region climate change policies have highest frequencies of the terms ‘wildlife’ and ‘biodiversity’. An exception is the Rwanda NAPA which does not mention the term ‘wildlife’. However, National Development Plans and Visions, for example South Africa’s National Development Plan Vision for 2030, do not even mention the terms ‘wildlife’ or ‘biodiversity’. However, other national visions such as the Botswana Vision 2016 mention ‘wildlife’ but do not mention ‘biodiversity’.

## Conclusion

Our findings show that international climate change policies like the UNFCCC and Kyoto Protocol do not mention the terms ‘biodiversity’ and ‘wildlife’. However, regional policies, in particular the EAC, have taken more advanced steps to mainstream biodiversity and wildlife issues in their climate change policies. The southern Africa region (SADC) has not mainstreamed climate change agenda in biodiversity and wildlife policies. However, the national climate policies from the SADC region address biodiversity and wildlife issues. Generally, for the selected countries, there is a disparity in the depth of coverage pertaining to biodiversity management issues between the National Development Plans/Visions and the national climate change strategies. Future studies should focus on reviewing the institutional programmes and projects for selected countries.
